# A bibliometric analysis of the global impact of metaproteomics research

**DOI:** 10.3389/fmicb.2023.1217727

**Published:** 2023-07-05

**Authors:** AbdulAziz Ascandari, Suleiman Aminu, Nour El Houda Safdi, Achraf El Allali, Rachid Daoud

**Affiliations:** ^1^African Genome Center, Mohammed VI Polytechnic University, Ben Guerir, Morocco; ^2^Department of Biochemistry, Ahmadu Bello University, Zaria, Nigeria

**Keywords:** metaproteomics, metagenomics, microbiome, bibliometric analyses, field weighted citation impact

## Abstract

**Background:**

Metaproteomics is a subfield in meta-omics that is used to characterize the proteome of a microbial community. Despite its importance and the plethora of publications in different research area, scientists struggle to fully comprehend its functional impact on the study of microbiomes. In this study, bibliometric analyses are used to evaluate the current state of metaproteomic research globally as well as evaluate the specific contribution of Africa to this burgeoning research area. In this study, we use bibliometric analyses to evaluate the current state of metaproteomic research globally, identify research frontiers and hotspots, and further predict future trends in metaproteomics. The specific contribution of Africa to this research area was evaluated.

**Methods:**

Relevant documents from 2004 to 2022 were extracted from the Scopus database. The documents were subjected to bibliometric analyses and visualization using VOS viewer and Biblioshiny package in R. Factors such as the trends in publication, country and institutional cooperation networks, leading scientific journals, author’s productivity, and keywords analyses were conducted. The African publications were ranked using Field-Weighted Citation Impact (FWCI) scores.

**Results:**

A total of 1,138 documents were included and the number of publications increased drastically from 2004 to 2022 with more publications (170) reported in 2021. In terms of publishers, *Frontiers in Microbiology* had the highest number of total publications (62). The United States of America (USA), Germany, China, and Canada, together with other European countries were the most productive. Institution-wise, the Helmholtz Zentrum für Umweltforschung, Germany had more publications while Max Plank Institute had the highest total collaborative link strength. Jehmlich N. was the most productive author whereas Hettich RL had the highest h-index of 63. Regarding Africa, only 2.2% of the overall publications were from the continent with more publication outputs from South Africa. More than half of the publications from the continent had an FWCI score ≥ 1.

**Conclusion:**

The scientific outputs of metaproteomics are rapidly evolving with developed countries leading the way. Although Africa showed prospects for future progress, this could only be accelerated by providing funding, increased collaborations, and mentorship programs.

## 1. Introduction

The development of “omics” technologies has revolutionized the field of molecular biology by enabling the analysis of biological systems at various molecular levels. The omics approaches which include genomics, transcriptomics, proteomics, and metabolomics focus on a specific aspect of molecular biology, allowing researchers to gain a comprehensive understanding of biological systems. Of course, these have contributed to numerous technological advancements across a wide range of fields (Seyhan and Carini, 2019).

One special field of omics named “meta-omics” has enabled the understanding of the microbial world. It involves the use of high-throughput sequencing technologies to investigate the collective genomic and functional potential of microbial communities. Using meta-omics, complex microbial communities (which cannot be cultured and studied in isolation) can be explored (Zhang et al., 2019; Mauger et al., 2022).

The different subfields of meta-omics (metagenomics, metatranscriptomics, metaproteomics, and metabolomics) work hand-in-hand to fully characterize the microbial population. In brief, metagenomics involves sequencing of DNA fragments in a sample, without the need for isolation and cultivation of individual microbes while metatranscriptomics focuses on the study of the RNA transcripts expressed by the microbial community, providing insights into the active functional pathways and metabolic processes of the community (Shakya et al., 2019; Maghini et al., 2021). Metaproteomics and metabolomics, on the other hand, involve the study of proteins and small molecules produced by microbial communities, providing insights into the metabolic, functional, and biochemical pathways of the community (Nephali et al., 2020; Armengaud, 2023). Hence, new biological molecules of significant importance can be identified, thereby unlocking new opportunities for biotechnology and environmental sciences (Zhang et al., 2019; Tiwari and Taj, 2020).

While metagenomics provides information on the potential functional capabilities of microbial communities, metaproteomics can confirm which functions are actively being performed (Schiebenhoefer et al., 2019). As many studies are focusing on metagenomics, coupling these studies with metaproteomics can address some of the limitations of metagenomics (Issa Isaac et al., 2019; Zheng et al., 2020). For example, metagenomics can be limited by the quality and completeness of the genomic data obtained from environmental samples, while metaproteomics can provide direct evidence of the proteins that are being expressed by microbial communities in the sample (Nowrotek et al., 2019; Renu et al., 2019).

Promising metaproteomics research has been published in recent years. The increase in the publication output could be associated with the technological advancements made in instrumentation (Bailey et al., 2019). One of the advancements is the development of Nano-Liquid chromatography (LC) techniques that are more advantageous than the conventional LC techniques, as well as the advent of high-resolution mass spectrometry (MS) that enables the identification and quantification of tens of thousands of peptides and proteins per sample (Vargas Medina et al., 2020). These have contributed to the understanding of proteins and their significance in different conditions (Hardouin et al., 2021).

However, despite the remarkable advancements, metaproteomics is still in its infancy, and its full potential has not been realized, particularly in developing nations (Hamdi et al., 2021). For instance, few studies in omics were conducted in Africa, most of which are collaborative with the developed world. Although these could be attributed to the lack of funding from the government and private sectors, the limited expertise in use of the advanced technologies, bioinformatics analysis, and interpretation of the generated data could be a limiting factor (El Jaddaoui et al., 2020; Iskandar et al., 2021).

Interestingly, the field of metaproteomics has existed for so long, and its impact on the functional characterization of the microbial population is far-reaching. Many publications have widely used the technology to determine the abundance, diversity, and activity of proteins in different research domains (Chandran et al., 2020; Rane et al., 2022). To mention a few, metaproteomics has been used in studies such as examining the microbiome composition and function (Kumar et al., 2021), comparative proteomics in health and disease states (Calabrese et al., 2022), evaluating the effects of environmental toxins (Jiao et al., 2022), and insights into metabolic pathways (Armengaud, 2023). Furthermore, the technique has been applied in the development of novel biomarkers for medical diagnostics as well as a better understanding of the function proteins play in host-pathogen interactions (Moreira et al., 2021). It is important to note that several reviews have discussed improvements made in metaproteomics (Schiebenhoefer et al., 2019; Andersen et al., 2021; Hardouin et al., 2021; Salvato et al., 2021; Bahule et al., 2022). However, there is scanty information concerning the state-of-art in the field of metaproteomics compared to other omics.

In this article, we conducted a bibliometric analysis in the field of metaproteomics. This kind of analysis has been conducted in other fields to have a full grasp of the achievements made (Md Khudzari et al., 2018; Yu et al., 2018; Kushairi and Ahmi, 2021; Wang et al., 2021). The specific objectives are to assess the current state of metaproteomic research on a global scale, evaluate its significance and potential impact, and specifically examine the contribution of Africa in the research field. The study aims to provide insights into the overall trends, collaboration patterns, and key research areas in metaproteomics globally while highlighting Africa’s research output and its potential role in advancing the field. By understanding the global landscape and Africa’s contributions, this study can inform future research directions, identify potential collaborations, and promote the growth of metaproteomics research in Africa.

## 2. Materials and methods

### 2.1. Data source and search strategy

The bibliometric analysis was conducted by searching for publications on metaproteomics in the Scopus database. Initially, a search was conducted on the Web of Science and Scopus databases to draw a comparison between their outputs. It was discovered that the Scopus database contained the majority of publications. The Scopus database is a comprehensive abstract and indexing database, offering extensive coverage across a wide range of subjects. It provides access to a significant number of international journals, ensuring global coverage and facilitating the inclusion of research from different geographic regions (Baas et al., 2020; Zhang et al., 2020). Keywords were selected by initially conducting a review of the relevant literature to identify commonly used terms related to our research topic. The MeSH (Medical Subject Headings) term of “Metaproteomics,” thus “Metaproteogenomic” was identified and the keywords “Metaproteomic” and “Metaproteogenomic” were used to search the titles, abstracts, and keywords of the Scopus database in combination with the Boolean operator “OR.” The wild card “*” was used at the end of the search term to cater to all other variants of the term, that is, “metaproteomic, metaproteomics, metaproteogenomic, and metaproteogenomics.” Only English publications were considered for this work. Hence, the query string used is as follows: TITLE-ABS-KEY (metaproteomic* OR metaproteogenomic*) AND [LIMIT-TO (LANGUAGE, “English”)] AND [EXCLUDE (PUBYEAR, 2023)]. Consequently, the results starting from 2004 to 2022 were collected. Altogether, 1,144 publications of all literature types were retrieved and included in order to ensure the overall comprehensiveness of the metaproteomics research. The completeness of the bibliographic metadata was determined using Biblioshiny (Supplementary Figure 1). In the database, information on journals, publication date, authors’ institutions, countries, publication sources, abstract, citation frequency, keywords, and bibliographies was selected and subsequently downloaded in a CSV file. In order to clean the data, duplicates were searched and removed, and the remaining literature was used for subsequent analysis. To obtain more information, the author’s h-index and Scopus ID, in addition to the journal cite scores, were retrieved from the database. It is important to mention that, in order to eliminate possible bias caused by database updates, data searching and gathering were conducted on the same day (January 17, 2023).

### 2.2. Bibliometric analyses and data visualization

The bibliometric analysis was conducted according to previous studies (Md Khudzari et al., 2018; Ejaz et al., 2022). VOSviewer (due to its user-friendly interface, ability to handle large datasets, and intuitive visualization options) and the Biblioshiny package in R (Aria and Cuccurullo, 2017) were used for the analysis. The VOSviewer has been known to be useful in developing more sophisticated bibliometric maps (Yu et al., 2018, 2020). The combination of the software and the R package enables in-depth analyses that aid in a more holistic understanding of scholarly collaboration, research impact, and citation dynamics in specific studies (Ragazou et al., 2022).

Using VOSviewer (Version 1.6.18), Country cooperation network, Institutional collaboration network, and Keyword analysis were conducted. Briefly, after importing the CSV file into the software, the co-authorship option was chosen, and the unit of analysis was designated as “countries.” The maximum number of writers per document and the counting method were left as defaults, while threshold criteria were set to a minimum of five (5) papers per country and a minimum of one (1) citation. For the Institutional collaboration network, a thesaurus file was developed and imported into VOSviewer in order to harmonize and re-label synonymic institutional names. Thereafter, co-authorship was selected, and organizations were chosen as the unit of analysis, while the counting method and the maximum number of organizations per document were left as defaults. The threshold criteria were set at a minimum of three (3) documents and zero (0) citations per organization. The type of analysis chosen for the keyword analysis was co-occurrence, and the unit of analysis was Author keywords, with a minimum number of keyword occurrences of five (5). The default counting method was used. A thesaurus file was developed and imported into VOSviewer to re-label synonymic single words and congeneric phrases. Overall, VOSviewer was used to construct network maps for keyword analysis, as well as collaboration between countries and institutions.

For the Biblioshiny, the CSV file was uploaded for analysis of trends in publication, major document types, peer-reviewed scientific journals, country productivity, and distributions of the author’s productivity. The analyses were performed by clicking the tabs on the web interface as follows:

Trends in publication: To access information in relation to the trend in publication, the *Biblioshiny→ Overview→ Annual Scientific Production→ Table* tabs were clicked. The annual scientific production is based on the total number of publications within each year.

Major document types: The *Biblioshiny → Overview→ Main information* tabs were clicked to have the information on the document types and their quantity.

Peer-reviewed scientific journals: Information related to the Leading Scientific Journals was accessed by clicking *Biblioshiny → Sources → Most relevant Sources* tabs.

Country Productivity: For the Country’s Productivity, *Biblioshiny → Authors → Countries’ Scientific Production* tabs were selected. Information on the total citation count and the average article citations of the countries was obtained by clicking *Biblioshiny → Authors → Most Cited Countries* tabs. The map of international collaboration between the countries was obtained by clicking *Biblioshiny → Social Structure → Countries’ Collaboration World Map* tabs.

Distribution of Author’s Productivity: Information concerning the leading Authors in Metaproteomics research was accessed by clicking *Biblioshiny → Authors → Most Relevant Authors* tabs. The author’s productivity period was ascertained by clicking *Biblioshiny → Authors → Author’s Production Over-Time → Plots* tabs. The analysis of the Author’s productivity using Lotka’s law was performed by clicking the tabs Biblioshiny → Authors → Lotka’s law.

Trending topics: To analyze trending terms based on the Keywords, the trend topics plot was generated by clicking the *Biblioshiny → Documents → Trend topics → Plot* tabs.

### 2.3. Sub-analysis of African publication trends and ranking using FWCI parameter

The emergence of African contributions to the field of metaproteomics was investigated by assessing the distribution and the overall trends in publication activities. To achieve this, data from publications related to Africa were extracted and analyzed using Excel. The ranking of the publications was conducted using the FWCI parameter from Scopus (Zanotto and Carvalho, 2021).

## 3. Results

### 3.1. Publications retrieval and screening process

**Figure 1:**
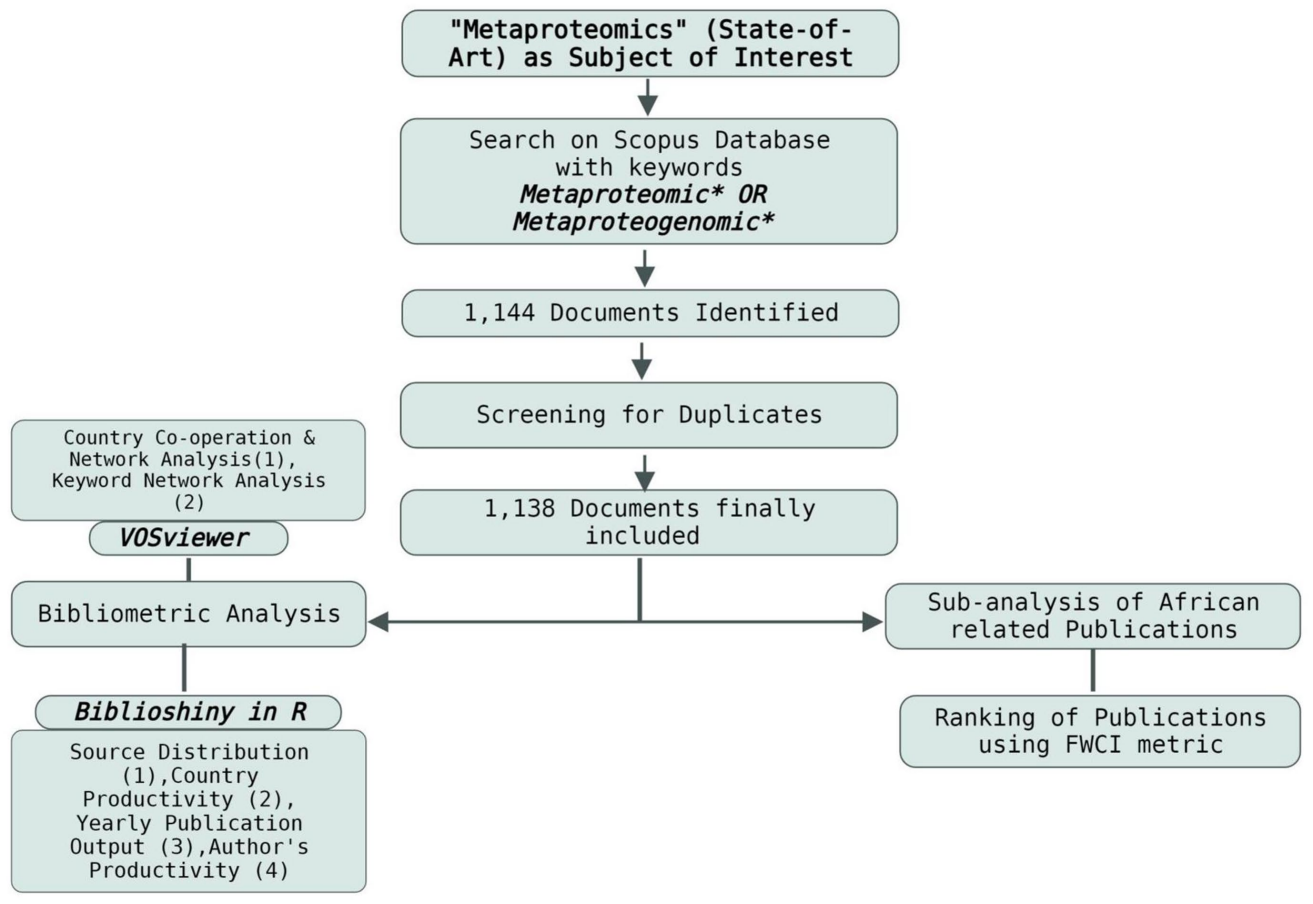
Workflow of documents retrieval process and analyses.

From the Scopus database, 1,144 publications were collected. After screening for duplicates, the number of articles was reduced to 1,138. A bibliometric analysis of the 1,138 publications on metaproteomics obtained from the database was conducted to understand the global impacts of metaproteomics research. The publications included research articles, reviews, conference papers, editorials, book chapters, and others. The workflow of the retrieval process is indicated in Figure 1.

### 3.2. The trend in publication output and major document types

**Figure 2:**
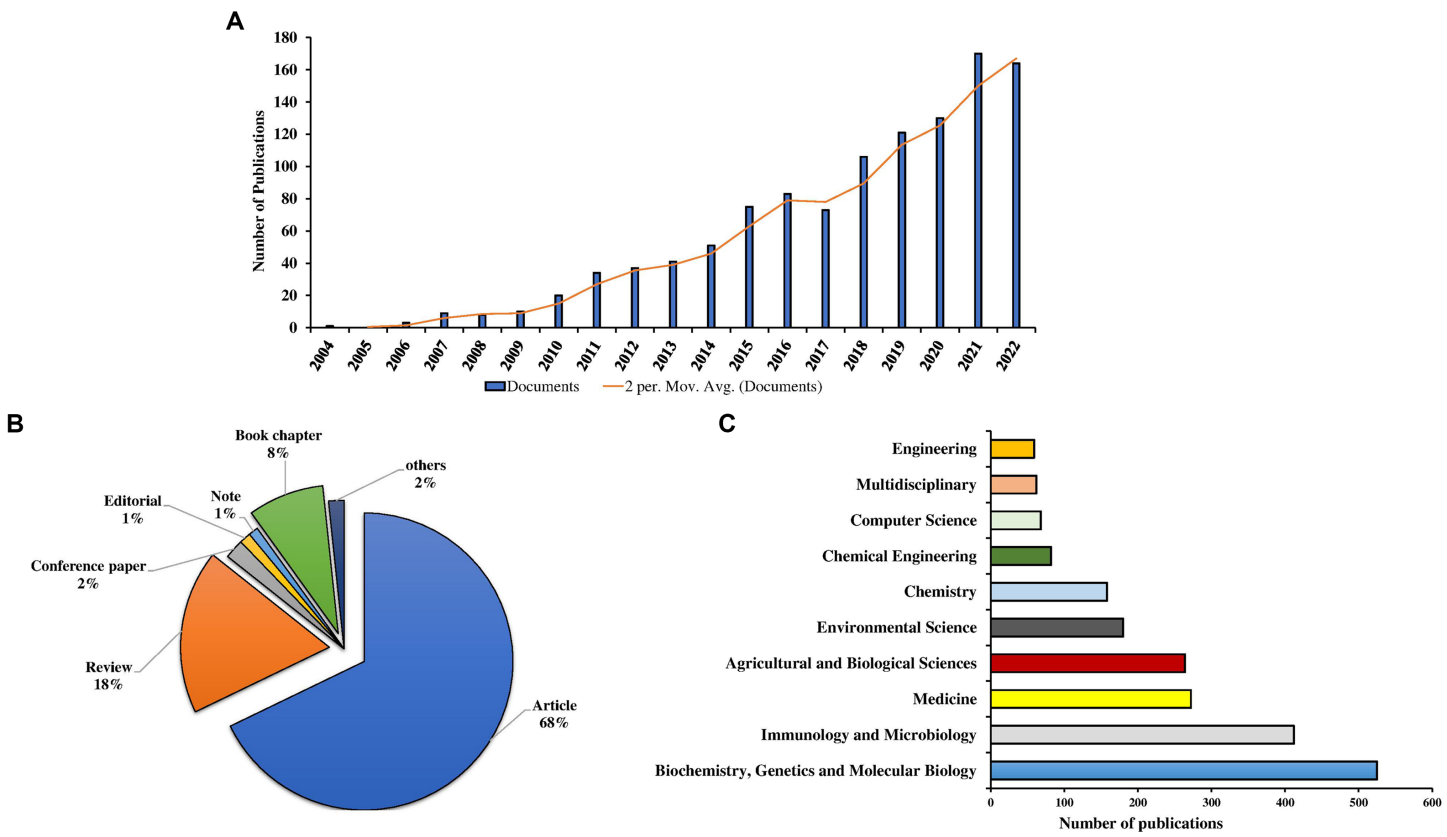
Global publication trend **(A)**, document types **(B)**, and global leading subject areas **(C)** in metaproteomics research.

The analysis revealed that the number of publications on metaproteomics has been increasing steadily since the early 2000s. Initially, studies on the topic started in 2004 with a single study. Afterwards, a slow increase in the number of publications was observed from 2006 to 2009. A significant rise in the number of publications was observed from 2010 onwards (Figure 2A). From 2010 onwards, there was a steady rise in the number of publications. Although a high number of publications (164; 14.4%) were recorded in 2022, there was more publication output in 2021 (170; 14.9%). Moreover, there was no publication record found in 2005. Overall, the estimated annual growth rate (EAGR) of the publications computed from the Biblioshiny package was 37.25%.

In terms of the document types (Figure 2B), the majority of the publications were research articles (772; 68%), followed by reviews (203; 18%) and book chapters (94; 8%). Others, including conference papers, editorials, and notes, account for 3.95%. Information gathered from the Scopus database revealed that the majority of the publications are all open access (678; 59.6%).

### 3.3. Leading subject areas and peer-reviewed scientific journals

The publications on metaproteomics included in the Scopus database cover 23 subject areas. The Major areas with the most densely distributed publications are Biochemistry, Genetics, and Molecular Biology with 525 published documents representing 46.13% of the total number of scholarly works (Figure 2C). Other subject areas with publications include Immunology and Microbiology (412; 36.20%), Medicine (272; 23.90%), Agricultural and Biological science (264; 23.19%), Environmental science (180; 15.82%), Chemistry (158; 13.88%), Chemical Engineering (82; 7.21%), Computer Science (68; 5.98%), Multidisciplinary (62; 5.45%), and Engineering (59; 5.19%) (Figure 2C).

The present finding investigated the major Journal houses that published the metaproteomic research, and among the top 10, *Springer Nature* (86; 7.56%) and *Wiley-Blackwell* (77; 6.77%) were the top publishers (Table 1). The *American Chemical Society (ACS)*, *Elsevier*, *Frontiers Media S.A.*, and the *Public Library of Science* were also among them (Table 1).

**Table 1:** The top 10 most productive journals that published metaproteomics research.

Sources	No. of publications	CiteScore (2021)	Publisher
Frontiers in Microbiology	62	8.2	Frontiers Media S.A.
Journal of Proteome Research	50	7.7	American Chemical Society
Proteomics	49	8.1	Wiley-Blackwell
Microbiome	37	24.5	Springer Nature
ISME Journal	32	18.2	Springer Nature
Environmental Microbiology	28	8.2	Wiley-Blackwell
Journal of Proteomics	25	7.0	Elsevier
Applied and Environmental Microbiology	24	7.8	American Society for Microbiology
PLoS One	20	5.6	Public Library of Science
Nature Communications	17	23.2	Springer Nature

In terms of the top 10 journals, *Frontiers in Microbiology* has the highest number of publications (62; 5.45%), followed by the *Journal of Proteome Research* (50; 4.39%), *Proteomics* (49; 4.31%), *Microbiome* (37; 3.25%), and *ISME Journal* (32; 2.88%). Conversely, while assessing the Journal’s CiteScore from the Scopus database (2021 report), *Microbiome* has the highest score of 24.5, followed by *Nature Communications* (23.2), and *ISME Journal* (18.2) (Table 1).

### 3.4. Country productivity and cooperation network

Looking at the country’s productivity, the top 20 most productive countries (in terms of publications) are the USA (359), Germany (257), China (172), and Canada (90). These countries have the highest total citations (>1,504), in addition to other countries like Spain (1,728), Sweden (1,620), and the United Kingdom (1,414). Contrariwise, Switzerland, Finland, and the UK have high average article citations of 147.3, 58.0, and 56.6, respectively (Table 2).

**Table 2:** The top 20 most productive countries involved in metaproteomics research.

Country	Total number of publication	Total number citation	Average number of article citations
United States	359	8,201	36.45
Germany	257	5,575	38.45
China	172	4,202	30.45
Canada	90	1,504	26.3
United Kingdom	86	1,414	56.56
Italy	83	1,363	25.72
India	81	622	14.14
Spain	77	1,728	37.57
France	76	1,086	27.85
Belgium	57	751	28.88
Australia	52	1,330	42.90
Denmark	51	390	28.14
Netherlands	49	197	32.50
Sweden	35	1,620	18.45
Austria	34	577	48.08
Switzerland	32	203	147.27
Finland	27	522	58.00
Brazil	21	294	16.73
Japan	21	266	42.00
Norway	19	184	26.60

**Figure 3:**
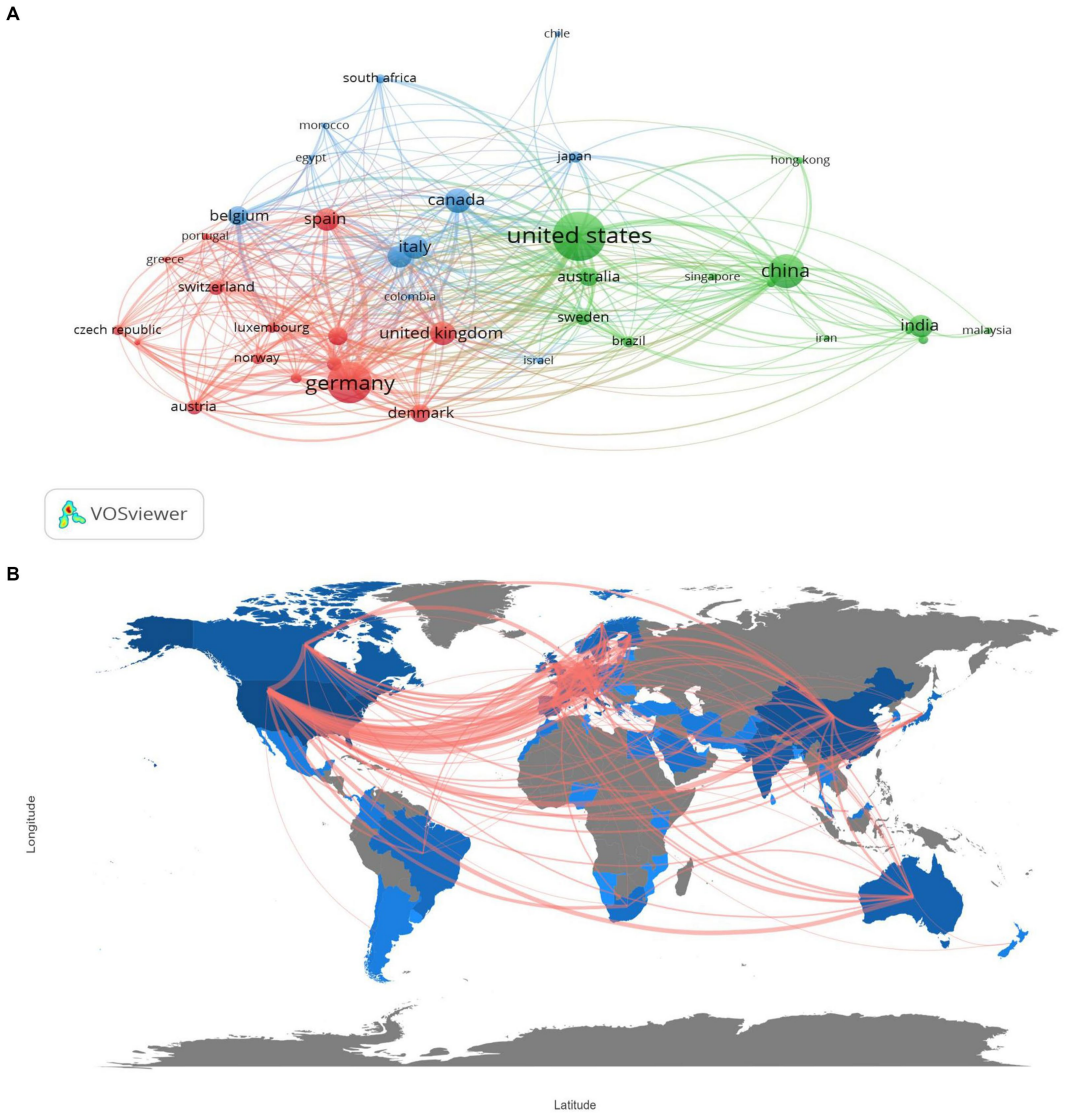
Network of country co-operation **(A)**, and worldwide map indicating international collaborations **(B)** in metaproteomics research.

The map and network of international and country cooperation (Figures 3A,B) demonstrate close collaboration among countries or regions involved in metaproteomics research. In the first cluster, the USA is the most affiliated country, linked to 38 countries or territories with a total link strength of 330, while China, as the second, is affiliated with 28 countries with a link strength of 119. Germany also has a total of 30 links and a total link strength of 334, followed by the UK in the second cluster. The final cluster includes Italy and Canada with more collaboration networks.

In terms of African countries, Morocco had the highest number of collaborations (14 links), followed by Egypt (13 links) and South Africa (11 links), with total link strengths of 20, 18, and 28, respectively.

### 3.5. Top institutions and distribution of author’s productivity

**Figure 4:**
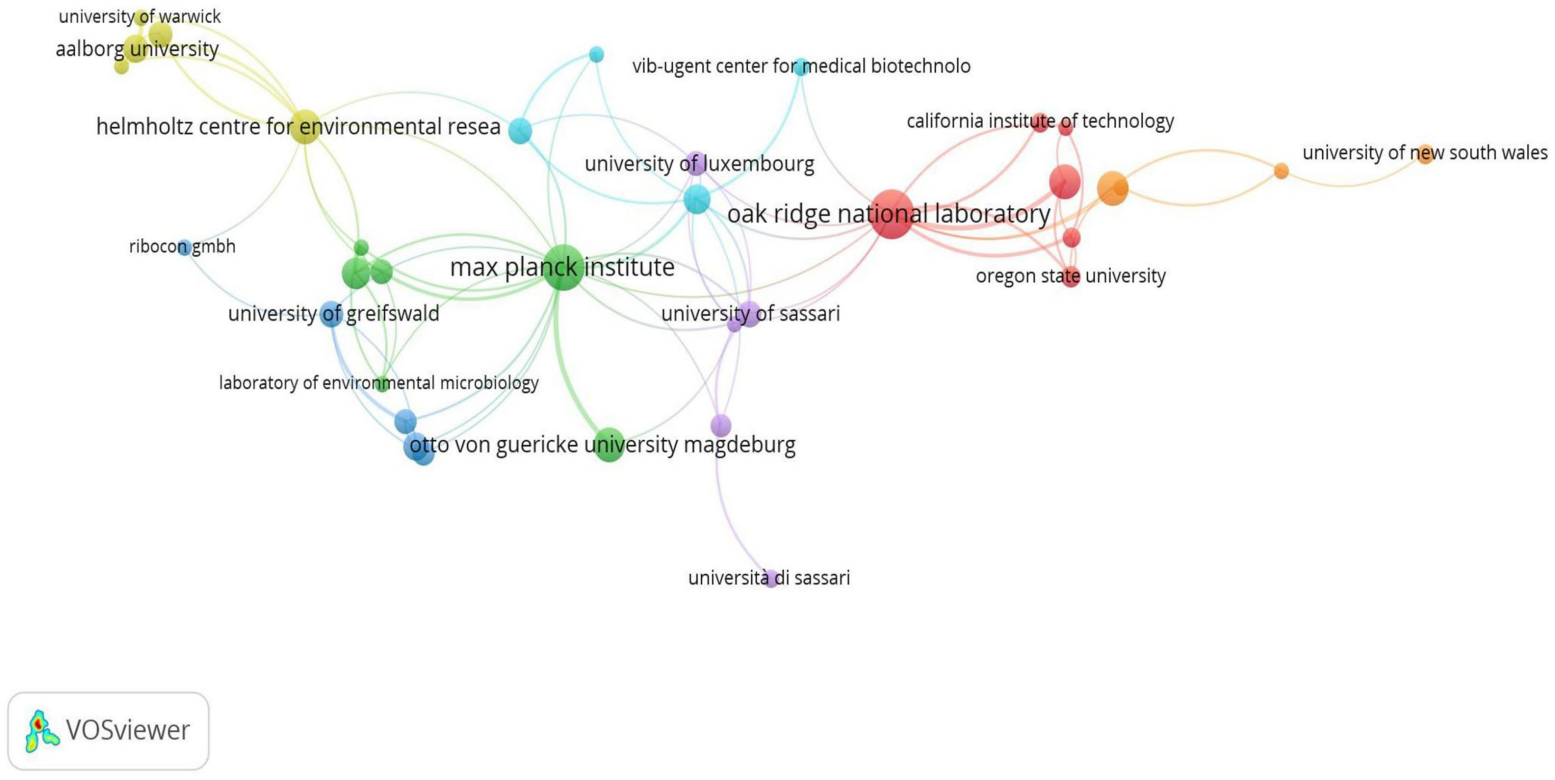
Institutional collaboration network in metaproteomics research.

Among the institutions, Helmholtz Zentrum für Umweltforschung, Germany (83; 7.29%), Oak Ridge National Laboratory, USA (59; 5.18%), Otto von Guericke University of Magdeburg, Germany (46; 4.04%), and Universität Greifswald, Germany (42; 3.69%) are the top institutions (Table 3). In terms of institutional collaborations, the Max Planck Institute, Oak Ridge National Laboratory, and Helmholtz Centre for Environmental Research have the highest total link strength of collaborations (Supplementary Figure 2). Figure 4 depicts the collaboration network among these institutions.

**Table 3:** The top 10 most productive institutions involved in metaproteomics research.

Institution	Documents published	Country
Helmholtz Zentrum für Umweltforschung	83	Germany
Oak Ridge National Laboratory	59	United States
Otto von Guericke University of Magdeburg	46	Germany
Universität Greifswald	42	Germany
Max Planck Institute for Dynamics of Complex Technical Systems	40	Germany
Universiteit Gent	39	Belgium
Pacific Northwest National Laboratory	37	United States
The University of Tennessee, Knoxville	35	United States
University of Ottawa	34	Canada
Universität Leipzig	31	Germany

**Figure 5:**
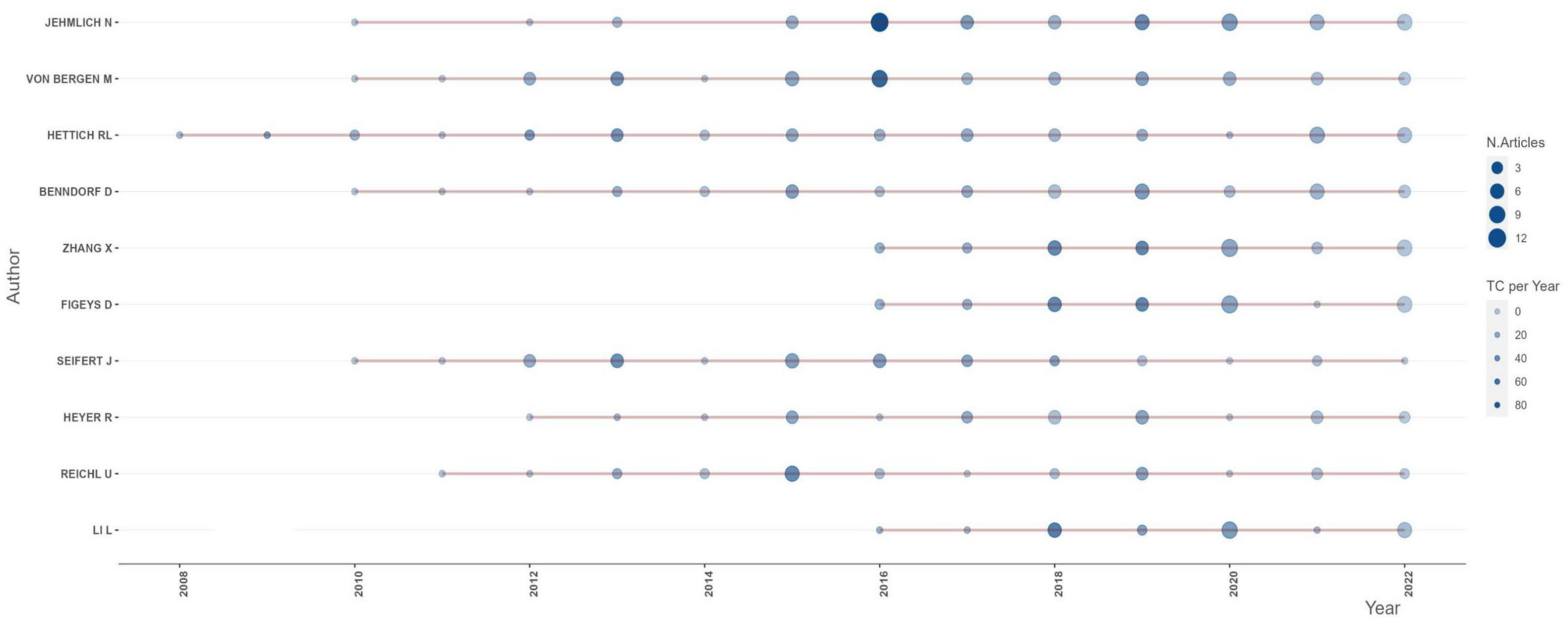
Yearly productivity of top authors involved in metaproteomics research.

In terms of authorship, the major authors (top 20) are from Germany, Canada, the United States of America (USA), France, Spain, Belgium, and Luxembourg, in that order. Among them, the author “Jehmlich N” from Helmholtz Centre for Environmental Research, Germany, has the highest publication record with 61 articles. His productive period spans from 2010, and he currently holds an h-index of 41 (Table 4; Figure 5). “Von Bergen M.” and “Hettich R.L.”, affiliated with the Helmholtz Centre for Environmental Research, Germany, and the Oak Ridge National Laboratory, USA, respectively, are ranked second and third. Similar to Jehmlich N., “Von Bergen M.” started his productive period in 2010 and currently holds an h-index of 56, while “Hettich R.L.” has an index of 63 with his first publication in 2008. Other authors with their h-index and productivity period are shown in Table 4 and Figure 5, respectively. An analysis of Lotka’s law reveals a skewed distribution of productivity among the authors contributing to metaproteomics research (Supplementary Figure 3).

**Table 4:** Top 20 prolific authors with high publication outputs in metaproteomics research.

Authors	Publications	Scopus ID	Current h index	Current affiliated institution	Country
Jehmlich N	61	24366846200	41	Helmholtz Zentrum für Umweltforschung	Germany
Von Bergen M	52	6603254363	56	Helmholtz Zentrum für Umweltforschung	Germany
Hettich RL	47	7006786519	63	Oak Ridge National Laboratory	United States
Benndorf D	43	55933825600	23	Otto von Guericke University of Magdeburg	Germany
Zhang X	36	57192503794	28	Université d'Ottawa, Faculté de Médecine	Canada
Figeys D	34	7005139451	57	Université d'Ottawa, Faculté de Médecine	Canada
Seifert J	34	35230690700	39	Universität Hohenheim, Stuttgart	Germany
Heyer R	29	55263328800	15	Leibniz-Institut für Analytische Wissenschaften	Germany
Reichl U	28	6602727388	49	Otto von Guericke University of Magdeburg	Germany
Li L	27	57194857863	16	Université d'Ottawa, Faculté de Médecine	Canada
Ning Z	27	25642980100	27	University of Ottawa,Faculté de Médecine	Canada
Mayne J	24	7004815175	26	Université d'Ottawa, Faculté de Médecine	Canada
Bastida F	23	14057630300	38	CEBAS- CSIC, Centro de Edafología y Biología Aplicada del Segura	Spain
Riedel K	23	57202754539	15	Universität Greifswald, Greifswald	Germany
Griffin TJ	21	7202249196	43	University of Minnesota Twin Cities	United States
Schallert K	21	57193336359	7	Leibniz-Institut für Analytische Wissenschaften	Germany
Armengaud J	20	6603746656	42	Université Paris-Saclay, Gif-sur-Yvette	France
Martens L	20	15923262500	56	Universiteit Gent, Ghent	Belgium
Tanca A	20	34769263100	25	Università degli Studi di Sassari, Sassari	Italy
Wilmes P	20	57207607143	47	University of Luxembourg	Luxembourg

### 3.6. Keyword analysis and current trending topics

**Figure 6:**
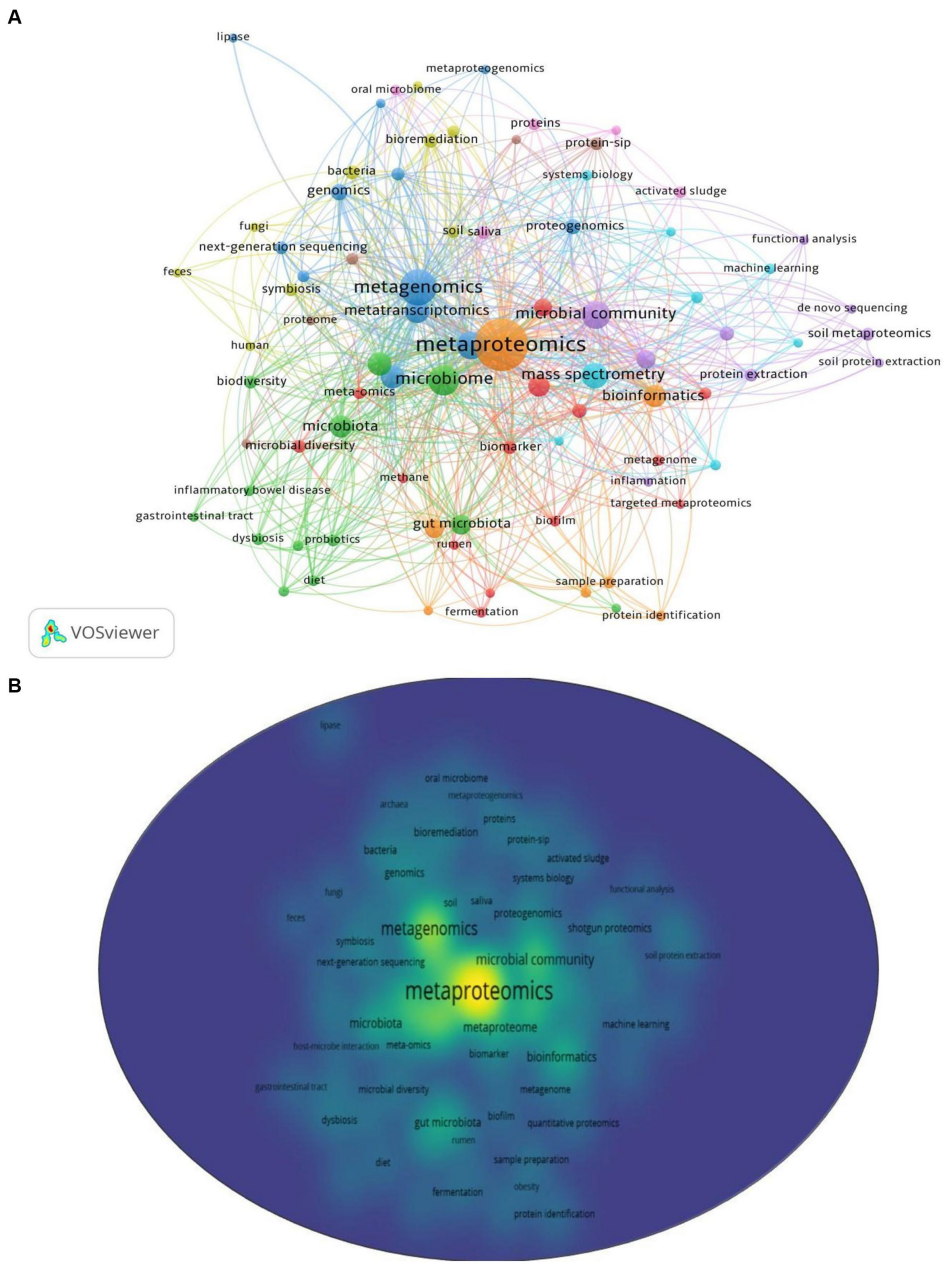
Analyses of keywords used in metaproteomics research **(A)** with their density visualization **(B)**.

Among 1,138 publications, a total of 2,459 keywords were identified. After re-labeling synonymous single words and consolidating similar phrases, 82 keywords met the threshold of a minimum of 5 occurrences for mapping in VOS viewer (Version 1.6.18). As shown in Figures 6A,B, nine clusters were formed and categorized in Table 5 to clearly indicate the selected and leading keywords in each cluster. Clusters 1 to 3 contained more than 10 keywords, while clusters 4 to 9 had fewer than 10 keywords. Cluster 1 had the highest number of selected keywords (14), followed by Cluster 2 (13) and Cluster 3 (12).

**Table 5:** Keyword clusters and leading keywords in metaproteomic research.

Cluster	Number of selected keywords	Selected keywords	Leading keywords
1	14	16S rRNA gene sequencing, anaerobic digestion, biofilm, biogas, biomarker, fermentation, lc–ms/ms, meta-omics, metagenome, metaproteome, methane, microbial diversity, rumen	Metaproteome (41 occurrences, 27 links, 51 total link strength)
2	13	Biodiversity, cystic fibrosis, diet, dysbiosis, gastrointestinal tract, gut microbiota, inflammatory bowel disease, microbiome, microbiota, obesity, omics, probiotics, short-chain fatty acids	Microbiome (115 occurrences, 60 links, 274 total link strength)
3	12	Genomics, lipase, metabolomics, metagenomics, metaproteogenomics, microorganisms, next-generation sequencing, proteogenomics, proteomics, rhizosphere, transcriptomics	Metagenomics (173 occurrences, 61 links, 447 total link strength)
4	9	Archaea, bacteria, biodegradation, bioremediation, feces, fungi, human, soil, symbiosis	Bacteria (15 occurrences, 19 links, 42 total link strength)
5	9	*de novo* sequencing, functional analysis, inflammation, microbial community, microbiology, protein extraction, shotgun proteomics, soil metatranscriptomics, soil protein extraction	Microbial community (93 occurrences, 46 links, 212 total link strength)
6	8	Human gut microbiome, liquid chromatography, machine learning, mass spectrometry, peptide identification, quantitative proteomics, system biology, tandem mass spectrometry	Mass spectrometry (72 occurrences, 44 links, 207 total link strength)
7	7	Covid-19, bioinformatics, gut microbiome, human microbiome, metaproteomics, protein identification, sample preparation	Metaproteomics (480 occurrences, 79 links, 937 total link strength)
8	5	Environmental microbiology, host–microbe interaction, metabolism, Protein-SIP, proteome	Protein-SIP (14 occurrences, 13 links, 28 total link strength)
9	5	Activated sludge, oral microbiome, proteins, saliva, wastewater treatment	Saliva (13 occurrences, 17 links, 41 total link strength)

Some leading keywords encountered in each cluster, based on their degree of occurrence, included metaproteome (Cluster 1; 41 occurrences; 27 links; 51 total link strength), microbiome (Cluster 2; 115 occurrences; 60 links; total link strength 274), metagenomics (Cluster 3; 173 occurrences; 61 links; 447 total link strength), bacteria (Cluster 4; 15 occurrences; 19 links; 42 total link strength), microbial community (Cluster 5; 93 occurrences; 46 links; 212 total link strength), mass spectrometry (Cluster 6; 72 occurrences; 44 links; 207 total link strength), metaproteomics (Cluster 7; 480 occurrences; 79 links; 937 total link strength), Protein-SIP (Cluster 8; 14 occurrences; 13 links; 28 total link strength), and saliva (Cluster 9; 13 occurrences; 17 links; 41 total link strength).

**Figure 7:**
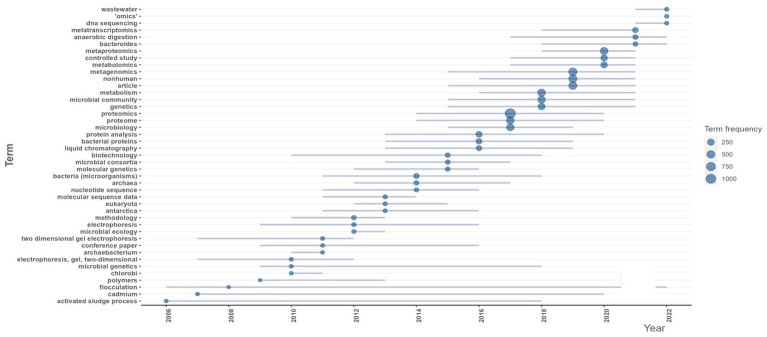
Global trending topics in metaproteomics research.

In order to analyze the trending terms based on the keywords, the Trend topic plot was constructed (Figure 7). The size of the circles shows the frequency of the term, and the length of the lines shows how long it has been studied (Abafe et al., 2022). From the plot, wastewater, omics, and DNA sequencing are the trending topics in recent years. The three most commonly used terms are proteomics (*f* = 1110), metagenomics (*f* = 564), and metaproteomics (*f* = 412) (Figure 7).

### 3.7. Publication analyses from Africa

**Figure 8:**
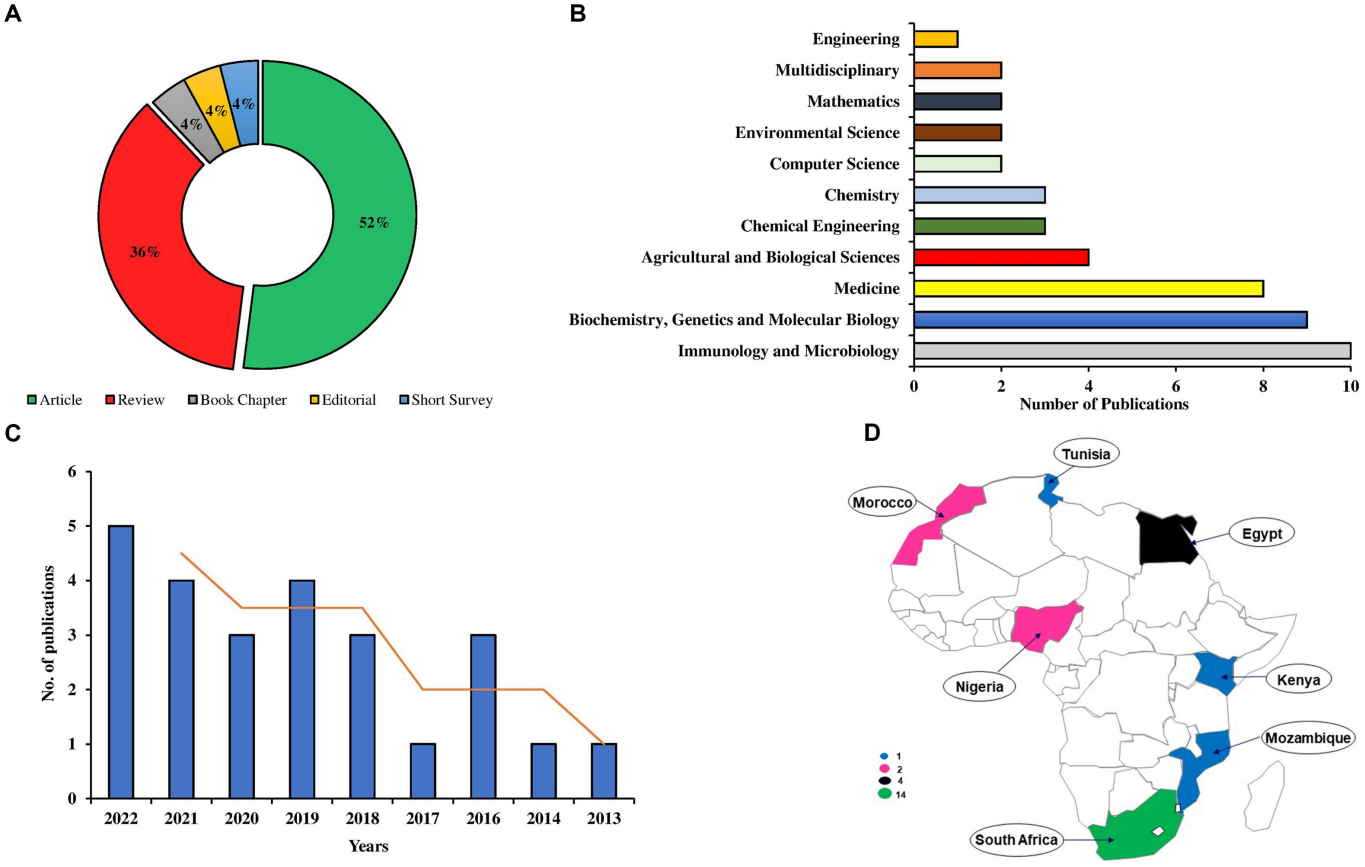
Analyses of the African metaproteomics publication including the document types **(A)**, leading subject areas **(B)**, yearly publication trend **(C)**, and geographical distribution of publication output **(D)**.

The Scopus database retrieved 13 articles, 9 reviews, 1 book chapter, 1 editorial, and 1 short survey related to metaproteomics research in Africa (Figure 8A). Accordingly, the major research areas that utilized metaproteomics include Immunology and Microbiology (10 publications), Biochemistry, Genetics, and Molecular Biology (9 publications), as well as Medicine (8 publications) (Figure 8B).

Looking at the trend of publication, it was observed that metaproteomics research in Africa began in 2013 (Figure 8C). From 2013 onwards, there has been an exponential growth in the publication outputs, with an EAGR of 19.58% (Supplementary Figure 4).

The countries in Africa, including South Africa (14; 56%) and Egypt (4; 16%), have the highest number of publications, while Nigeria and Morocco have 2 publications, accounting for 8% each. Other countries with a single publication in metaproteomics are Kenya, Mozambique, and Tunisia (Figure 8D). Supplementary Figure 4 provides additional information about the general characteristics of the African publications. Furthermore, we previously highlighted the country cooperation networks of three African countries: Morocco, Egypt, and South Africa, in Figure 3A. The top 10 journals for African publications are presented in Supplementary Table 1, with *Frontiers in Cellular and Infection Microbiology* and *Microbiome* emerging as the leading journals.

**Figure 9:**
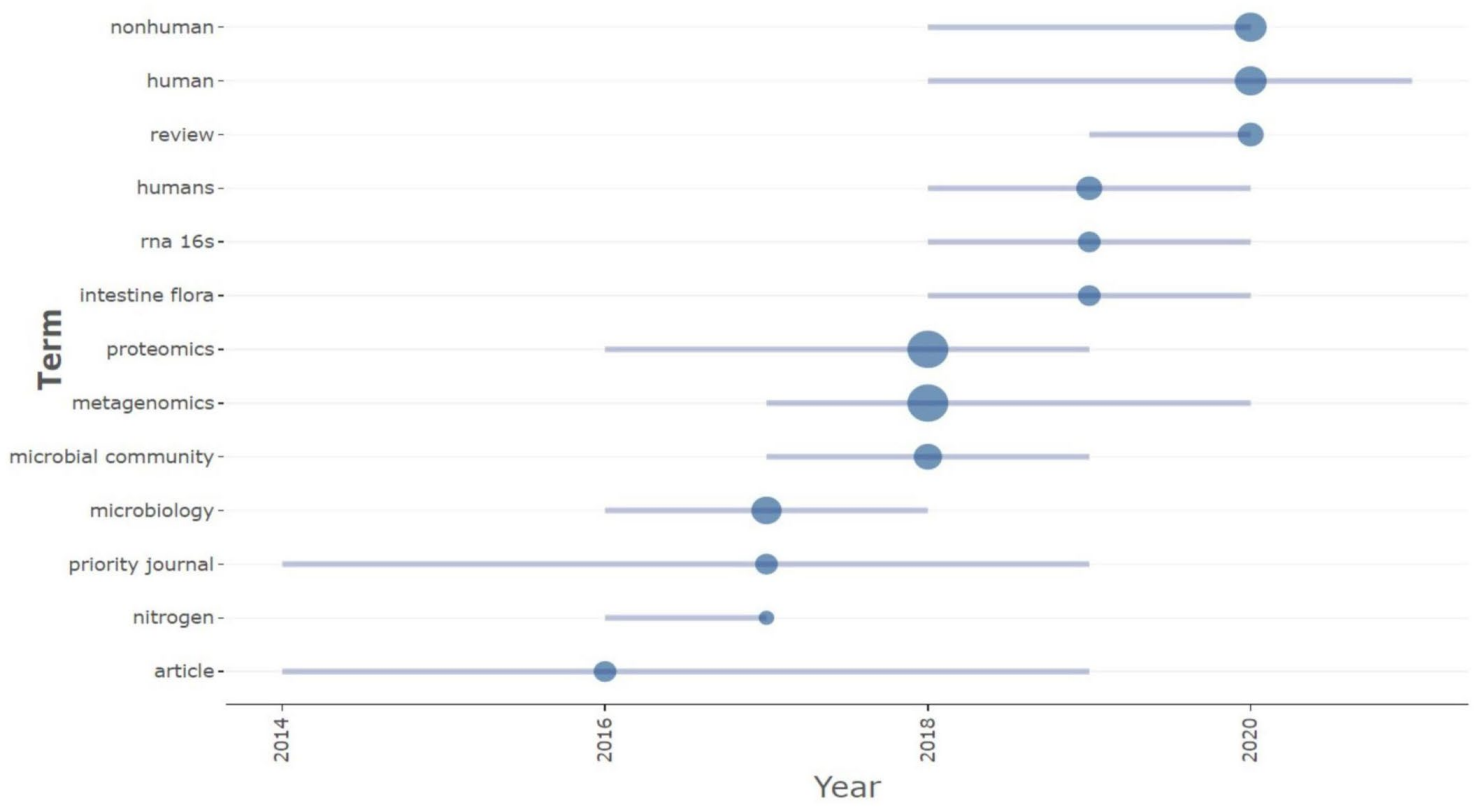
Trending topics in metaproteomics research in Africa.

For the keyword analyses, a minimum threshold of two (2) occurrences was set, resulting in the identification of 10 keywords that met the criteria. The majority of the keywords are metaproteomics, metagenomics, metatranscriptomics, and microbiome. In terms of the network, the leading keywords are metabolites (Cluster 1), metaproteomics (Cluster 2), and metagenomics (Cluster 3). According to the Trend topic plot, it is evident that non-human and human research, along with review writing, are currently trending topics in the field. In concordance with the global trend, proteomics (*f* = 17) and metagenomics (*f* = 17) are the most commonly used terms (Figure 9).

### 3.8. Ranking publications from Africa using the field-weighted citation impact (FWCI) scores

The African publications were ranked using FWCI (Zanotto and Carvalho, 2021). About 52% of publications had an FWCI score of 1 or higher, indicating a positive impact (Table 6). For instance, most of the publications from South Africa, Egypt, Morocco, Kenya, Tunisia, and Nigeria have FWCI scores ≥1. One study from Morocco and a study from Mozambique have scores <1, respectively.

**Table 6:** Field weighted citation impact (FWCI) of African-affiliated publications in metaproteomics.

Ranking	Author and their publication year	Total citations	Principal affiliated African institution	Country	Field weighted citation impact (FWCI)
1	Blackburn and Martens (2016)	14	Institute of Infectious Disease and Molecular Medicine, Department of Integrative Biomedical Sciences, University of Cape Town, Cape Town, South Africa	South Africa	3.5
2	Lau et al. (2016)	117	Department of Microbial Biochemical, and Food Biotechnology, University of Free State, Bloemfontein, 9301, South Africa	South Africa	3.2
3	Malan-Muller et al. (2018)	89	Department of Psychiatry, Faculty of Medicine and Health Sciences, Stellenbosch University, Tygerberg, 7600, South Africa	South Africa	2.4
4	Liu et al. (2019)	25	International Livestock Research Institute (ILRI), Mazingira Centre for Environmental Research and Education, Box 30709, Nairobi, 00100, Kenya	Kenya	1.9
5	Moussa et al. (2022)	4	Department of Clinical Pathology, School of Medicine, Mansoura University, Mansoura, Egypt	Egypt	1.8
6	Andrés-Barrao et al. (2016)	36	Agricultural Genetic Engineering Research Institute (AGERI), Agricultural Research Center (ARC), Giza, Egypt	Egypt	1.5
7	Abdool Karim et al. (2019)	28	Centre for the AIDS Programme of Research in South Africa (CAPRISA), University of KwaZulu-Natal, Durban, South Africa	South Africa	1.4
8	Kantor et al. (2017)	29	Centre for Bioprocess Engineering Research, Department of Chemical Engineering, University of Cape Town, Rondebosch, 7701, South Africa	South Africa	1.3
9	Ugya et al. (2019)	8	Department of Environmental Management, Kaduna State University, Kaduna, Nigeria	Nigeria	1.2
10	Ekwanzala et al. (2021)	1	Department of Environmental, Water and Earth Sciences, Tshwane University of Technology, Pretoria, South Africa	South Africa	1.2
11	Daffonchio et al. (2013)	24	Laboratory of Microorganisms and Active Biomolecules, University of Tunis El Manar, Tunis, Tunisia	Tunisia	1.2
12	Magnabosco et al. (2018)	15	Department of Microbial, Biochemical and Food Biotechnology, University of the Free State Bloemfontein, Free State, 9300, South Africa	South Africa	1.1
13	Lahlali et al. (2021)	7	Plant Pathology Unit, Department of Plant Protection, Ecole Nationale d’Agriculture de Meknes, BP S/40, Meknes, 50001, Morocco	Morocco	1.0
14	Alisoltani et al. (2020)	9	Division of Medical Virology, Department of Pathology, University of Cape Town, Cape Town, 7925, South Africa	South Africa	0.8
15	Guo et al. (2018)	13	DST/NRF Centre of Excellence for Biomedical Tuberculosis Research, SAMRC Centre for Tuberculosis Research, Division of Molecular Biology and Human Genetics, Faculty of Medicine and Health Sciences, Stellenbosch University, Cape Town, 7505, South Africa	South Africa	0.8
16	Moosa et al. (2020)	19	Africa Health Research Institute, Durban, South Africa	South Africa	0.6
17	Okeke et al. (2021)	6	Department of Biochemistry, Faculty of Biological Sciences, University of Nigeria, Nsukka, 410001, Enugu State, Nigeria	Nigeria	0.5
18	Gunnigle et al. (2014)	15	Centre for Microbial Ecology and Genomics (CMEG), Department of Genetics, University of Pretoria, South Africa	South Africa	0.5
19	Lukhele et al. (2020)	10	Nanotechnology and Water Sustainability Research Unit, College of Science Engineering and Technology, University of South Africa, Science Campus, Johannesburg, South Africa	South Africa	0.4
20	Bahule et al. (2022)	1	Center of Studies in Science and Technology (NECET), Universidade Rovuma, Niassa branch, Lichinga, Mozambique	Mozambique	0.3
21	Sehli et al. (2021)	3	Department of fundamental sciences, School of Medicine, Mohammed VI University of Health Sciences, Casablanca, Morocco	Morocco	0.3
22	Ezzeldin et al. (2019)	4	Proteomics and Metabolomics Unit, Department of Basic Research, Children’s Cancer Hospital Egypt, Cairo, 57357, Egypt	Egypt	0.1
23	Delgado-Diaz et al. (2022)	0	Division of Medical Virology, Department of Pathology, University of Cape Town, Cape Town, 7925, South Africa	South Africa	0.0
24	Chigorimbo-Murefu et al. (2022)	0	Divisions of Medical Virology, Institute of Infectious Diseases and Molecular Medicine, University of Cape Town, Cape Town, South Africa	South Africa	0.0
25	Hirtz et al. (2022)	0	Higher Institute of Engineering and Technology, Alexandria, New Borg AlArab City, 21934, Egypt	Egypt	0.0

## 4. Discussion

Metaproteomics is a rapidly growing field of research that seeks to understand the complex microbial communities that inhabit the world (Kumar et al., 2021). To better understand the impact of this research area, tracking and analyzing its scientific outputs will be paramount in identifying emerging trends and key contributors in the field. To achieve this goal, we conducted a bibliometric analysis of more than 1,100 publications on metaproteomics obtained from the Scopus database. As the Web of Science is also commonly utilized for this type of study, we started our study with a comparative analysis, which revealed that 97.3% of the total extracted publications from the Scopus database are shared with the Web of Science. Only 2.7% of the publications (31 in total) are unique to the Web of Science database (statistics of these 31 publications have been included in the Supplementary material). From the analysis, information on the publication patterns and collaboration structures, among others was studied. A similar statistical analysis was adopted recently in order to understand the trends and the focus of the link between gut microbiota and type 1 diabetes (Guo et al., 2023).

Since 2004, the yearly trend in publication outputs on metaproteomics has been increasing with a steady growth rate starting from 2010. This increase, in part, could be attributed to the increased interest of scientists in identifying novel proteins for several biotechnological applications from microbial communities (Purohit et al., 2020; Mishra et al., 2021). Although there is not much difference in publication output between 2021 and 2022, the higher numbers in 2021 could be attributed to several notable advancements in metaproteomics tools within the year. For instance, the use of single-cell proteomics to study microbial communities and the development of methods for analyzing post-translational modifications in metaproteomics samples (Taylor et al., 2021; Armengaud, 2023), in addition to COVID-19 pandemic which spurred interest in using the technique to study the human microbiome and its role in infectious diseases (He et al., 2021; Sun et al., 2022). Overall, the global EAGR suggests that, under normal circumstances, the number of publications in metaproteomics is expected to increase by 37% over time. The abundance of research articles in this field suggests that many authors are actively engaged in experimental research and eager to advance our understanding by conducting thorough analyses of their findings. Their contributions are likely to shed new light on the intricate workings of protein chemistry in a particular phenomenon, potentially leading to significant breakthroughs and progress.

Based on the leading subject areas, most of the publications are related to Biochemistry, Genetics, and Molecular Biology as well as Immunology and Microbiology. This indicates that metaproteomics is a promising field that helps scientists gain a more comprehensive understanding of biological systems. Using the techniques of metaproteomics, an enhanced understanding of genetics, as well as possible changes that might occur in disease states, could be achieved (Henry et al., 2022). Also, some publications were on Agriculture and Environmental sciences, enabling the design of strategies that ensures agricultural productivity and environmental sustainability (Chandran et al., 2020; Bahule et al., 2022).

Analysis of the top 10 journals reveals that all of them are of high quality, with both Q1 or Q2 rankings and impact factors above 4. When examining the journals’ CiteScore, it becomes apparent that both Microbiome and Nature Communications have a substantial impact on the metaproteomics research community. The CiteScore could influence the decision of some authors when selecting a journal for their work (Roldan-Valadez et al., 2019). Overall, based on the findings, it could be asserted that researchers in the field of metaproteomics will be inclined to publish their findings in these top 10 journals.

In terms of country productivity and cooperation network, Europe and America have the highest total number of publications. Individually, the USA has the highest number of total citations followed by Germany and China, indicating that these countries have a high overall impact on the research output. Contrary to the total citation counts, Switzerland, Finland, and the UK exhibit high average article citations, indicating the production of high-quality research by these countries (Roldan-Valadez et al., 2019). As the total citation measures quantity (Tahamtan and Bornmann, 2019), the average citation measures quality and determines scientific contribution as well as plays a role in advancing a specific field. For collaboration, it appears that the USA, Germany, China, Italy, and Canada are the most connected countries in addition to some other European countries. The collaboration networks observed could be attributed to scientific advancements and investment in research and development (R&D) in these countries (El Jaddaoui et al., 2020; Guo et al., 2023).

Consistent with the above analysis, the institutions from Europe and America have the highest publication outputs, with German institutions taking the lead. Generally, German institutions have a long history of scientific excellence, and they have been at the forefront of many scientific breakthroughs. Specifically, in the case of metaproteomics research, German institutions have been able to leverage their expertise in proteomics and microbiology to develop methods and techniques in metaproteomic analyses (Schiebenhoefer et al., 2019). In terms of collaboration networks, there are more links among German institutions, with the Max Planck Institute having the highest collaboration. This observation is not surprising, as the institution has been observed to be frequently involved with metaproteomic research. To sum it up, the high publication outputs in the aforementioned top 10 institutions from Europe (Germany) and America may be due to a variety of factors, including access to funding, a high level of expertise and specialized knowledge, successful collaborations with other researchers and institutions, and strategic research priorities that are in line with the institution’s goals.

The diversity of research partners, a high proportion of foreign postgraduates and visiting academics, and robust research funding are all possible contributors to the dynamism of international collaboration and increased publication outputs (Roldan-Valadez et al., 2019). The top 20 authors with the highest publication records are primarily from Europe and America. The foremost author among them serves as the group leader for Microbiome Biology at the Helmholtz-Center for Environmental Research in Germany. The key areas of interest for this author’s research are the study of microbiome biology in terrestrial microbial communities, metaproteomic studies, and both qualitative and quantitative microbial proteomics. In particular, the author is known for their work in identifying key microbial players using protein-SIP, a cutting-edge technique in the field of microbiology. The author’s first publication, titled “Phylogenetic and proteomic analysis of an anaerobic toluene-degrading community,” examined, for the first time, a sulfate-reducing community grown with toluene as a carbon source by a combination of molecular genetics and proteomic techniques in order to uncover the physiological interplay in this microbial anaerobic community (Jehmlich et al., 2010). The author achieved the highest publication output in 2016, resulting in a total citation count of 87.5, and subsequently, in 2020, the author’s publication output amassed a total citation count of 30.75. As well, it is worth highlighting that one of the top 20 authors is Wilmes P., who is a notable figure in the field. In collaboration with Bond P.L., Wilmes P. proposed the term “metaproteomics” in their 2004 publication entitled “The application of two-dimensional polyacrylamide gel electrophoresis and downstream analysis to a mixed community of prokaryotic microorganisms” (Wilmes and Bond, 2004). This contribution to the scientific community has had a significant impact on the field of metaproteomics and serves as a testament to Wilmes P.’s expertise and innovative thinking. In terms of h-index, Hettich R.L. (the leader of the Bioanalytical Mass Spectrometry group at Oak Ridge National Laboratory) has the highest value of 63 among the top 20. The h-index used here measures the productivity and impact of a researcher’s publications (Roldan-Valadez et al., 2019). It also takes into account engagements in secondary research, which could influence its value. An assessment of the author’s productivity in this field reveals that it adheres to Lotka’s law, which claims that a small percentage of authors produce the majority of output in a given field (Kushairi and Ahmi, 2021). This information could be useful for developing strategies to support and encourage the productivity of all authors in the field, regardless of their current level of output.

Keyword analyses offer a comprehensive overview of the research area’s trajectory and themes (Donthu et al., 2021). Based on the keyword analyses, Cluster 1 had the highest number of selected keywords, implying that it contains keywords frequently used in the context of metaproteomic research (Donthu et al., 2021). In terms of leading keywords, metaproteomics had the highest occurrence, followed by metagenomics and other omics. This is unsurprising as the omics fields are often coupled together in order to have a thorough understanding of the microbial world (Kumar et al., 2021; Jiang et al., 2022).

Further the keyword clusters generated give an overview of the thematic research hotspots. For instance Cluster 1 sheds light on the interest in understanding microbial processes and diversity in biotechnology and the environment while the terms in Cluster 2 are related to metaproteomics research aimed at understanding the importance of gut microbiota in human health. Clusters 3 and 4 on the other hand indicate interest in the concept of molecular components and interactions within biological systems as well as understanding the role of metaproteomics in investigating the soil microbiome and biodegradation and bioremediation processes. Further the other clusters concentrated on techniques for analyzing the protein expressions interactions and functional roles of microorganisms in the soil and gut microbiome in diseases such as COVID-19 as well as researching microbial communities and their functional proteins in activated sludge and wastewater systems. Some important diseases that have been researched using the metaproteomics approach in humans include obesity (Zhong et al., 2019; Biemann et al., 2021; Calabrese et al., 2022) cystic fibrosis (Debyser et al., 2019; Saralegui et al., 2022) inflammatory bowel disease (Moon et al., 2018; Zhang et al., 2018; Lehmann et al., 2019) and the oral microbiome (Jagtap et al., 2012; Bostanci et al., 2021). In addition to the research conducted on human diseases metaproteomics has also been extensively applied in the field of soil ecosystems – soil metaproteomics (Bastida et al., 2016b, 2018; Chourey and Hettich, 2018; Liu et al., 2019) – with specific focus on the rhizosphere (Renu et al., 2019; White et al., 2021). Other environmental phenomena such as bioremediation (Bastida et al., 2016a; Aishwarya et al., 2022) and biodegradation (Chang et al., 2018; Gunasekaran et al., 2022; Xie et al., 2022) has been explored

To further determine the metabolically active players in microbial communities, Protein SIP experiments are increasingly being conducted in the area of environmental microbiology. In addition, studies concerning fermentation or anaerobic digestion of samples from the environment such as biofilms or from wastewater treatment plants which leads to the production of biogas such as methane have been investigated (Joyce et al., 2018; Heyer et al., 2019; Lam et al., 2021; Chen et al., 2022).

Overall, the main instrumentation used in the analysis is tandem mass spectrometry. The peptides are initially separated using liquid chromatography, particularly in the LC–MS/MS procedure. Shotgun proteomics and quantitative proteomics are popular approaches employed to identify and quantify proteins (Balotf et al., 2022). The generated data are analyzed using bioinformatics tools in order to identify the proteomes in the studies.

Sub-analysis of African contributions in metaproteomics revealed that the continent contributed only 25 publications of the total 1,138. Among the 54 countries in Africa, only 7 countries engage in metaproteomic research. As previously mentioned, a lack of infrastructure, funding, and expertise (El Jaddaoui et al., 2020) could contribute to these low statistics. Therefore, considering the significant scientific impact of metaproteomics and omics in general, it is essential to redirect more research efforts toward Africa to characterize the microbial diversity on the continent (Cowan et al., 2022). Remarkably, the positive EAGR observed suggests that research efforts in the continent are increasing, although more has to be done in order to accelerate the growth. In terms of country collaboration, the collaboration network in the continent is relatively weak compared to global networking. Among the countries, only South Africa was noticed to establish research partnerships with the advanced countries.

Interestingly, in comparison with the major publishers, the publications from the African continent are also found in high-impact journals. Likewise, the top keywords used in Africa are comparable to those used globally, with metaproteomics ranking first. This word has links with the other omics terms. The trend plot portrays trending research topics related to non-human studies as well as human studies. Additionally, it is worth noting that review writing was highly predominant. To increase research output beyond review writing, it is recommended to establish more laboratories and research centers specifically dedicated to metaproteomics.

The FWCI calculates how frequently a given publication is cited relative to others in the same field. Given its normalization, it can be used to directly compare an article’s performance against those of other articles (even those in different subject areas). The FWCI ≥1 in some of the African publications indicates that the output performs exactly as expected by the global average, while the values <1 in some of the publications indicate underperformance in comparison to the global average (Zanotto and Carvalho, 2021). Hence, there is a need for African authors to develop means to improve their performance in order to increase the impact and visibility of their work.

To further emphasize, the insights gained from metaproteomics research have wide-ranging applications in fields such as medicine, agriculture, and environmental science. For instance, metaproteomics enables the taxonomic and functional analysis of complex microbial communities in various samples, allowing the quantification of proteins and providing a detailed understanding of cellular phenotypes at the molecular level. By leveraging metaproteomics, researchers can gain comprehensive insights into these complex biological systems and pave the way for advancements in various disciplines. Fernanda Salvato recently discussed, in detail, the extensive examples of metaproteomics-based approaches used to address a wide range of questions in diverse areas of biological research (Salvato et al., 2021).

## 5. Conclusion

In conclusion, the bibliometric analyses of more than 1,100 publications on metaproteomics from the Scopus database indicated an increasing trend of publications with a high percentage of open-access research articles. The majority of these publications fall within the field of Biochemistry, Genetics, and Molecular Biology, with *Microbiome* and *Nature Communications* emerging as the top publishers based on their CiteScore rankings. With respect to the author’s contribution and country productivity, Jehmlich N. was the most productive author, while the United States, Germany, and China were the most productive countries, collectively contributing to 69% of the total publications. Keyword analyses revealed metaproteomics and metagenomics as the highest co-occurring keywords in addition to other omics. Zooming in on Africa, only 2.2% of total publications came from the continent, with South Africa producing more publications. Based on the EAGR and the FWCI, more studies are required in the region in order to meet the global scale and contribute to the advancement of metaproteomics. To encourage this research area in Africa, some initiatives have begun to emerge. For instance, in Morocco, the Sharifian Phosphate Office (OCP) has funded a new proteomic platform at Mohammed VI Polytechnic University (RD is the principal investigator), which will help to advance the field of proteomics.

### 5.1. Recommendations

With the strides made by this emerging field, its global challenges cannot be overlooked. Some of the drawbacks in this field are in the areas of sample complexity and preparation, accurate protein database construction for microbial communities, false discovery rate assessments, annotation, and software integration (Issa Isaac et al., 2019; Rechenberger et al., 2019; Saito et al., 2019; Duong and Lee, 2023). Each of these challenges can affect the quality and reproducibility of metaproteomic results and addressing them will require collaborative efforts between researchers and software developers. By doing so, standardized protocols and tools that will improve data quality, accuracy, and reproducibility could be developed. In addition, the current bibliometric analysis has revealed a significant merit of metaproteomics in studying the spatiotemporal characterization of microbial communities at the functional level. Recently, Manuel Kleiner published a very interesting and detailed review that illustrates the diversity of questions that can be addressed solely through metaproteomics in the study of microbial communities, including those associated with plants and animals (Kleiner, 2019). This emerging area holds significant potential for advancing scientific knowledge, addressing biotechnological challenges, improving human health, and informing environmental management strategies. Further, to advance the field of metaproteomics in Africa, several steps can be taken. This includes prioritizing metaproteomics research by providing funding, equipment, and training opportunities. Also, encouraging collaboration between African and international researchers through joint projects, exchange programs, and workshops is essential. For instance, the International Metaproteomics Initiative (Van Den Bossche et al., 2021) is a commendable step toward advancing the field and promoting knowledge sharing. Lastly, support should be provided for publications in high-impact journals, travel grants, and mentoring programs to enhance the quality of research on the continent.

Overall, the findings of this study provide valuable insights that can be used to advocate for increased support, funding, and resources for metaproteomics research on a global scale, as well as specifically in Africa. These findings serve as a basis for informed decision-making by policymakers, enabling them to drive policies, allocate funds, and distribute resources in a manner that promotes the growth and impact of metaproteomics research. By harnessing the potential of metaproteomics, policymakers can address complex societal challenges by studying the intricate dynamics of microbial communities and their functions in various fields. Further, the application of metaproteomics in studying soil microbiomes, bioremediation, and biodegradation, for instance, can contribute to the development of policies related to sustainable agriculture, effective waste management, and environmental conservation.

### 5.2. Limitations of the study

Although the Scopus and Web of Science databases have been recognized as the largest searchable collection of citations and abstracts in the field of literature research, it would be interesting to incorporate other databases such as PubMed, and Cochrane into the present analysis subsequently. Also, language bias introduced in the selection of documents may inadvertently exclude contributions from non-English-published articles. Moreover, refining and expanding the search strategy to include a broader range of relevant terms, synonyms, and variations could help in capturing more documents for the bibliometric analysis.

## Data availability statement

The original contributions presented in the study are included in the article/Supplementary material, further inquiries can be directed to the corresponding authors.

## Author contributions

RD, AE, and AA: conceptualization. AA and SA: methodology. AA, SA, NS, AE, and RD: formal analysis, investigation, and writing—review and editing. AA, SA, and RD: writing—original draft preparation. RD, AA, AE, and SA: resources. RD: supervision. All authors contributed to the article and approved the submitted version.

## Conflict of interest

The authors declare that the research was conducted in the absence of any commercial or financial relationships that could be construed as a potential conflict of interest.

## Publisher’s note

All claims expressed in this article are solely those of the authors and do not necessarily represent those of their affiliated organizations, or those of the publisher, the editors and the reviewers. Any product that may be evaluated in this article, or claim that may be made by its manufacturer, is not guaranteed or endorsed by the publisher.

## Supplementary material

The Supplementary material for this article can be found online at: https://www.frontiersin.org/articles/10.3389/fmicb.2023.1217727/full#supplementary-material
