# Supplementary material for: A bibliometric analysis of the global impact of metaproteomics research
Source: Front Microbiol. 2023 Jul 5;14:1217727. doi: 10.3389/fmicb.2023.1217727 (PMC10354264; doi:10.3389/fmicb.2023.1217727)
Supplement: Supplementary file 1 [file Data_Sheet_1.docx]

Supplementary Material

A Bibliometric Analysis of the Global Impacts of Metaproteomics Research

AbdulAziz Ascandari ^1^, Suleiman Aminu ^1, 2^, Nour El Houda Safdi ^1^, Achraf El Allali ^1^, Rachid Daoud ^1*^

^1^African Genome Center, Mohammed VI Polytechnic University, Lot 660, Hay Moulay Rachid, Ben Guerir, Morocco

^2^Department of Biochemistry, Ahmadu Bello University, P.M.B. 1096, Zaria, Kaduna, Nigeria

*** Correspondence**Rachid Daoud
[rachid.daoud@um6p.ma](mailto:rachid.daoud@um6p.ma)

Achraf El Allali

[Achraf.ELALLALI@um6p.ma](mailto:Achraf.ELALLALI@um6p.ma)

Abdulaziz Ascandari

abdulaziz.ascandari@um6p.ma

Supplementary Figures and Tables

##
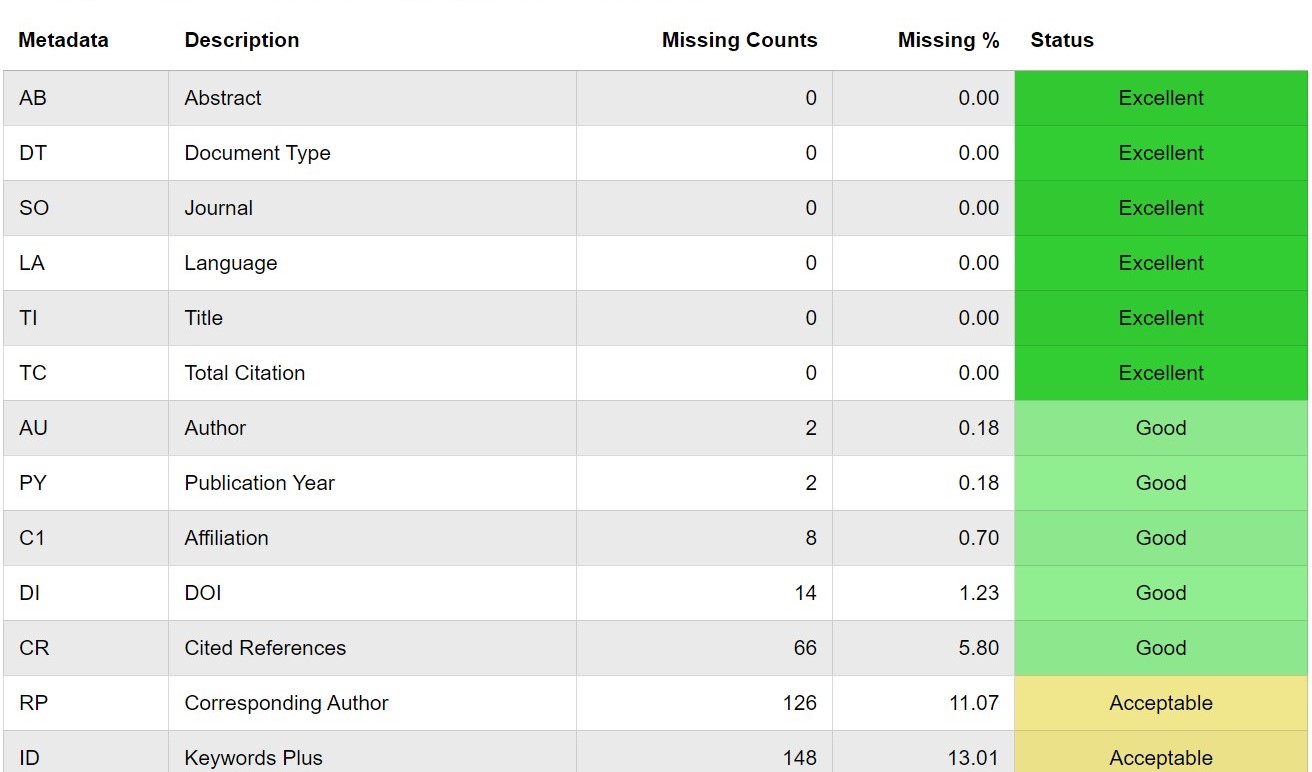
Supplementary Figures

**Supplementary Figure 1.** Quality control and the Completeness of Bibliometric Data collected from the Scopus Database


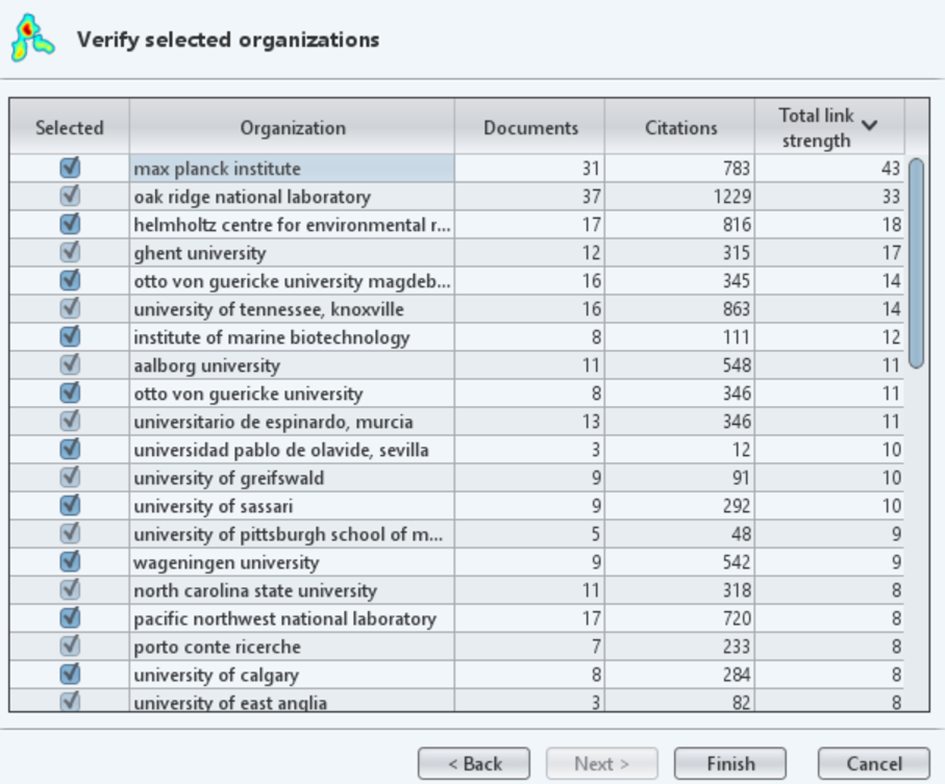


**Supplementary Figure 2.** Total Link strength of the institutional collaborations in Metaproteomics Research.

**Supplementary Figure 3.** Investigation of the Trend of Author’s Productivity using Lotka’s law


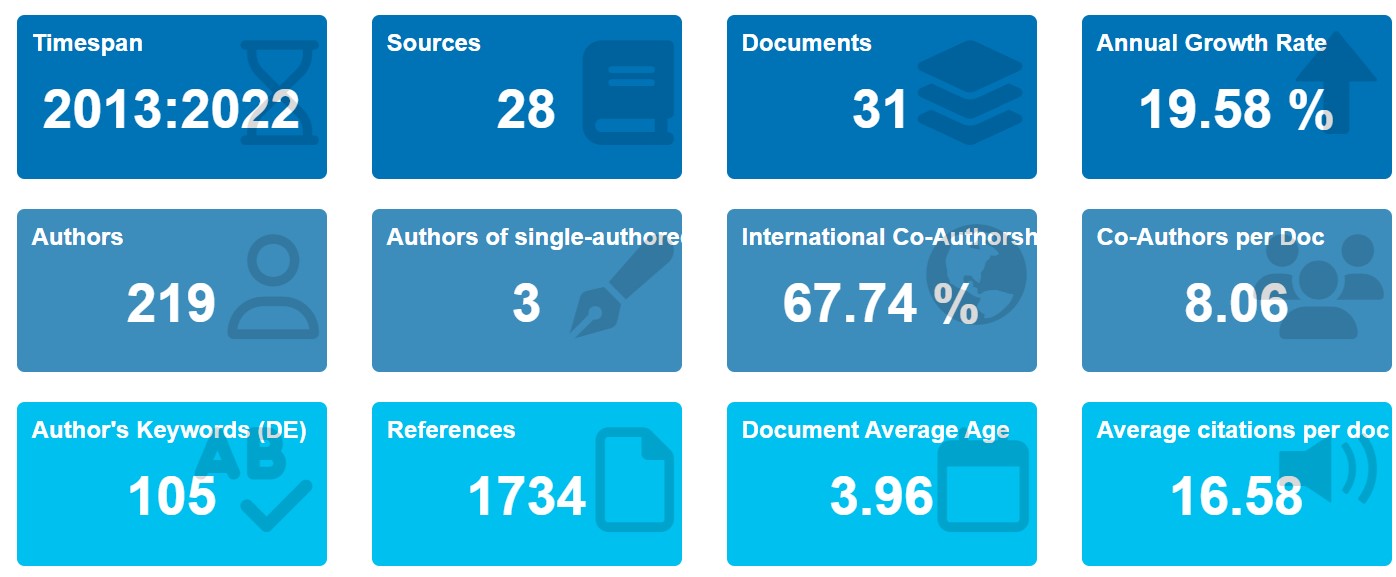


**Supplementary Figure 4.** Summary of General Information on Metaproteomics Publications from Africa

## Supplementary Tables

**Supplementary Table 1.** Top 10 Most Productive Journals Publishing Metaproteomics Research in Africa

| No. | Source | No. of documents | CiteScore 2021 | Publisher |
| --- | --- | --- | --- | --- |
| 1 | Frontiers In Cellular And Infection Microbiology | 2 | 5.9 | Frontiers Media S.A. |
| 2 | Microbiome | 2 | 24.5 | Springer Nature |
| 3 | ACS Omega | 1 | 5.2 | American Chemical Society |
| 4 | Applied Ecology And Environmental Research | 1 | 1.5 | Szent Istvan University |
| 5 | Bioinformatics | 1 | 13.4 | Oxford University Press |
| 6 | Bioinformatics And Biology Insights | 1 | 4 | Libertas Academica |
| 7 | Current Microbiology | 1 | 3.1 | Springer Nature |
| 8 | Environmental Science And Technology | 1 | 14.8 | American Chemical Society |
| 9 | Expert Review Of Proteomics | 1 | 6.8 | Taylor & Francis |
| 10 | FEMS Microbiology Ecology | 1 | 7 | Oxford University Press |

**Supplementary Table 2.** Summary Statistics of Publications unique to Web of Science comparative to Scopus database

| Time Span | 2004 - 2022 |
| --- | --- |
| Estimated Annual Growth Rate (EAGR) | 89.4% |
| **Documents** | |
| Number of documents | 31 |
| Average age of documents (years) | 5.5 |
| Average total citations per document | 18.19 |
| **Authors** | |
| Total number of Authors | 222 |
| Authors per document | 7.16 |
| **Keywords** | |
| Total Number of Author keywords | 45 |
| Highest Co-occurring Keywords | Metaproteomics (29 links; 34 total link strength; 7 occurrences) & metagenomics (19 links; 23 total link strength; 4 occurences) |
| **Document types retrieved** | |
| Article | 18 |
| Conference Paper | 10 |
| Review | 2 |
| Book chapter | 1 |
